# The Essential Role of Mitochondrial Dynamics in Viral Infections

**DOI:** 10.3390/ijms26051955

**Published:** 2025-02-24

**Authors:** Xujie Duan, Rui Liu, Wenjing Lan, Shuying Liu

**Affiliations:** 1 College of Veterinary Medicine, Inner Mongolia Agricultural University, Hohhot 010018, China; 2 Inner Mongolia Key Laboratory of Basic Veterinary Medicine, Inner Mongolia Agricultural University, Hohhot 010018, China; 3 Key Laboratory of Clinical Diagnosis and Treatment Technology for Animal Disease, Ministry of Agriculture, Hohhot 010018, China

**Keywords:** mitochondria, viral infection, mitochondrial dynamics, innate immunity, apoptosis

## Abstract

Mitochondria are dynamic organelles that play crucial roles in energy production, metabolic balance, calcium homeostasis, apoptosis, and innate immunity, and are key determinants of cell fate. They are also targets for viral invasion of the body. Many viral proteins target mitochondria, controlling mitochondrial morphology, metabolism, and immune response, thereby achieving immune evasion, promoting their proliferation, and accelerating the infection process. Mitochondrial quality control is key to maintaining normal physiological functions and mitochondrial homeostasis. Dysregulation of mitochondrial dynamics is closely related to the development of many diseases. New roles of mitochondrial dynamics in viral infection are constantly being discovered. Viruses change mitochondrial dynamics by targeting mitochondria to achieve a persistent state of infection. Currently, understanding of mitochondrial dynamics during viral infection is limited. Research on the impact of viral proteins on mitochondrial dynamics provides a foundation for investigating the pathogenesis of viral infections, the disease process, and identifying potential therapeutic targets. This review focuses on the connection between viral infection and mitochondrial dynamics and priority areas for research on virus-mediated mitochondrial immunity, provides insight into the regulation of mitochondrial dynamics by viruses targeting mitochondria, and explores potential means of mitochondrial-mediated control and treatment of viral diseases.

## 1. Introduction

Mitochondria are multifunctional organelles in eukaryotic cells and play a crucial role in maintaining the normal physiological functions of cells. Their main physiological function is to generate adenosine triphosphate (ATP) through oxidative phosphorylation (OXPHOS) to provide energy for the body. Other functions include fatty acid oxidation, participation in some forms of apoptosis, regulation of calcium ion balance, and synthesis and decomposition of metabolites. Abnormalities of these processes are known as mitochondrial dysfunction [1,2].

A complex set of mitochondrial quality control mechanisms within cells to maintain the integrity and normal function of the mitochondrial network and prevent mitochondrial dysfunction caused by intrinsic or external environmental stimuli [3]. Mitochondrial quality control (MQC) is an endogenous protective programme that maintains normal physiological functions and mitochondrial homeostasis through mitochondrial dynamics (such as regulating mitochondrial fusion and fission), mitophagy (the process of selective degradation of damaged mitochondria after being wrapped by autophagosomes and fusing with lysosomes), and mitochondrial biogenesis (the generation of new mitochondria from existing ones), protecting mitochondria from damage and preventing the accumulation of defective mitochondria [4,5]. Mitochondrial dynamics, including mitochondrial fusion and fission, are the core of MQC and key to ensuring the stability of various cellular functions. Mitochondrial dynamics coordinate cellular metabolism and influence complex cellular signalling processes, such as regulating cell pluripotency, division, differentiation, ageing, and death [6]. In addition, mitochondria play an important role in innate immunity, not only by generating mitochondrial damage-associated molecular patterns, but also by serving as a platform for innate immunity, carrying mitochondrial antiviral signalling protein (MAVS) and exerting its antiviral effect [7,8,9].

Viral parasitism in host cells leads to changes in host cell physiology, which can affect the mitochondrial network, resulting in mitochondrial dysfunction [10]. Mitochondria play a central role as a hub for innate immune signal transduction and metabolism, and they are important in the pathogenesis of viral infections. The role of mitochondrial dynamics in viral infection has attracted the attention of researchers in recent years. Many viruses have been shown to target mitochondrial dynamics-related proteins directly, disrupting the balance between mitochondrial fusion and fission, and influence immune function and apoptosis, achieving viral replication, assembly, and transmission, and inducing pathological changes related to infection [11,12,13,14].

This review aims to elaborate on the changes in mitochondrial dynamics induced by viral infection and discuss how viruses manipulate mitochondrial dynamics and resist mitochondrial antiviral responses to promote persistent infection and disease. Owing to limited understanding of the mechanisms of mitochondrial dynamics, the rules of viral manipulation of mitochondrial dynamics have not been clearly defined. This review summarises the recent literature on viral infection and mitochondrial dynamics, as well as how viruses use mitochondrial dynamics, functions, and signals to evade innate immune signals, thereby promoting viral replication and transmission. It also summarises the current status of research on antiviral drugs targeting mitochondrial dynamics in order to provide a source of reference for researchers in this field.

## 2. Mitochondria and Viral Infection

### 2.1. Structure and Function of Mitochondria

Mitochondria are highly structured organelles, consisting of four functional regions from the outside to the inside: the outer mitochondrial membrane (OMM), the intermembrane mitochondrial space, the inner mitochondrial membrane (IMM), and the matrix [15,16]. The OMM is porous, and pore-forming proteins such as voltage-dependent anion channels (VDACs) transport ions and uncharged small molecules, whereas large molecules are transported by special protein translocases [17]. The IMM is a tight diffusion barrier with selective permeability, allowing only specific ions and molecules to be transported by specific membrane transport proteins and serving as the site for OXPHOS [18]. The mitochondrial matrix provides a site for several enzymatic reactions, DNA replication, transcription, and protein biosynthesis, and generates a transmembrane electrochemical gradient to drive ATP synthesis [19]. Mitochondria also have a special structure, mitochondrial cristae, which have a distinct tubular inner structure and are formed by the inward folding of the IMM. The cristae can change their shape and capacity according to the energy demands of the cell. The cristae membrane contains most of the electron transport chain and ATP synthase and is the main site for bioenergetic conversion in mammals [20]. However, the OXPHOS process produces byproducts such as reactive oxygen species (ROS), which play important roles in cellular signal transduction and homeostasis. Excessive ROS damage mitochondrial proteins, lipids, and DNA stability [21]. The mitochondrial membrane potential (MMP) is the potential difference across the IMM and is a key determinant of the health status of cells. The MMP maintains normal OXPHOS and ATP production, and a decrease in MMP is associated with mitochondrial autophagy and apoptosis [22,23]. Mitochondria have their own genome and the complete equipment and ability for protein translation, with thousands of copies of circular mitochondrial DNA (mtDNA) in each cell, which replicate using their own transcription mechanism to participate in the formation of the IMM respiratory chain [24].

### 2.2. The Impact of Viral Infections on Mitochondria

Given that viruses lack the physiological mechanisms for energy metabolism and must parasitise living cells to perform their functions and considering that mitochondria control cell survival and apoptosis, mitochondria, as targets of viral invasion, undoubtably play a significant role in the host’s antiviral response. Viruses typically target mitochondrial function and structure, inducing changes in mitochondrial bioenergetics to provide energy and biosynthetic precursors for viral protein synthesis and assembly of mature viral particles, thereby gaining the ability to control host cell apoptosis, metabolic status, and evade the host immune response, to achieve viral particle replication [25,26]. Correspondingly, mitochondria are affected by viral targeting or changes in cellular physiological environments such as imbalances in calcium homeostasis, endoplasmic reticulum stress, oxidative stress, and hypoxia during viral infection, leading to mitochondrial dysfunction. After mitochondrial damage, mtDNA is released into the cytoplasm to mediate changes in cellular physiological and biochemical functions [27]. For example, herpes simplex virus 1 (HSV-1) infection of target cells changes in mitochondrial structure and function by inducing mitochondria to become rough, thin, and elongated, with shortened and thickened mitochondrial cristae, an increase in the number of endoplasmic reticulum–mitochondria contact (ERMC) sites, and an increase in mitochondrial Ca^2+^ content, ATP production, and reactivation [28]. Long-term infection with hepatitis B virus (HBV) causes cellular lesions, resulting in cytoskeletal disruption, cytoplasmic vacuolisation, and abnormal mitochondrial morphology, characterised by the disappearance of mitochondrial cristae, incomplete mitochondrial membranes, and mitochondrial swelling, suggesting that HBV may cause disease by inducing mitochondrial dysfunction [29]. Additionally, severe acute respiratory syndrome coronavirus 2 (SARS-CoV-2) binds to mitochondrial proteins, inhibiting the expression of mitochondrial genes encoded by the nucleus and mitochondria, leading to mitochondrial energy production disorders and immune response activation, and consequently causing multi-organ dysfunction [30]. Porcine reproductive and respiratory syndrome virus (PRRSV) infection leads to an increase in Ca^2+^ which induces mitochondrial fission and subsequently triggers mitophagy, and it utilizes cellular energy metabolism reprogramming to promote its own replication [31]. The above studies suggest that mitochondrial dysfunction may serve as a mechanism of viral infection pathogenesis. Viruses control mitochondrial function and energy metabolism to provide favorable conditions for their own replication, and restoring mitochondrial function may provide therapeutic ideas for such diseases. These studies indicate that mitochondrial dysfunction may serve as a pathogenic mechanism of viral infections, and that restoring mitochondrial function may have a therapeutic effect.

## 3. Mitochondrial Dynamics and Viral Infection

### 3.1. Mitochondrial Dynamics

Mitochondria are highly dynamic organelles that adjust their network according to cellular requirements, perform “quality control” on the organelles, and restore intracellular balance during energy depletion or after mitochondrial damage. During physiological processes such as cell differentiation and apoptosis, mitochondrial morphology changes are strictly regulated by mitochondrial dynamics. An imbalance between fission and fusion affects the normal function of mitochondria [32].

Fission and fusion are continuous processes. Mitochondria complete fusion extremely rapidly and rapidly exchange matrix metabolites. Severely damaged mitochondria are degraded and recycled through a strictly regulated process of mitophagy, preventing damaged or dysfunctional mitochondria from cycling in the pool of healthy mitochondria, and jointly maintaining the quality and function of mitochondria [33,34]. This relative balance mechanism of fission and fusion affects the immune response to viral infection. Many viruses strategically interfere with the MQC mechanism, inducing an imbalance between mitochondrial fission and fusion to affect mitochondrial morphology, quality, and function, thereby promoting viral invasion and influencing the cell’s immune defence against viruses.

#### 3.1.1. Mitochondrial Fusion

Mitochondrial fusion is an evolutionarily conserved process. In mammals, this process is mediated by three large dynamin-related GTPases, namely mitofusin 1 (Mfn1) and 2 (Mfn2), and optic atrophy protein 1 (Opa1). Due to the double-membrane structure of mitochondria, the mitochondrial fusion process is divided into two steps: outer membrane fusion and inner membrane fusion [35].

Mfn1 and Mfn2 are widely expressed in the OMM. Both have a transmembrane domain that places the N-terminal GTPase and heptad repeat 1 (HR1) domain in the cytoplasm and the C-terminal heptad repeat 2 (HR2) domain in the intermembrane space. Mfn1 and Mfn2 on adjacent mitochondria form homodimers or heterodimers through the oligomerisation of the HR2 structure, thereby binding two mitochondria together to mediate OMM fusion [36]. Knockout of either the *Mfn1* or *Mfn2* gene, or both, disrupts mitochondrial structure and induces severe cellular defects, including smaller and more fragmented mitochondria, lower MMP, weakened mitochondrial respiratory activity and ATP production, and inhibition of cell proliferation [37,38].

Opa1 has multiple subtypes related to the IMM. It mediates IMM fusion and maintains the structure of mitochondrial cristae. The long subtype of Opa1 (L-Opa1) is anchored in the IMM, whereas the short subtype of Opa1 (S-Opa1) has no obvious membrane anchoring. The combination of the long and short subtypes affects fusion activity [39]. One end of S-Opa1 dimerises with L-Opa1, and the other end interacts with the target membrane, whereas the other side of L-Opa1 is tethered to cardiolipin on the target membrane through guanosine triphosphate (GTP)-independent membrane tethering. GTP hydrolysis causes conformational changes, leading to the fusion of the two membranes. In contrast, the interaction of the same subtype of Opa1 mediates membrane tethering, thereby supporting the cristae structure [40]. The absence of Opa1 leads to mitochondrial fragmentation in mouse embryonic fibroblasts, and its overexpression induces mitochondrial elongation. Moreover, overexpression of Opa1 rescues mitochondrial fragmentation caused by the absence of Mfn2, but this rescue mechanism is ineffective for Mfn1, indicating that Opa1 functionally depends on Mfn1 [41].

#### 3.1.2. Mitochondrial Fission

Corresponding to the process of mitochondrial fusion, mitochondrial fission is a highly regulated dynamic process that results in one mitochondrion splitting into two or more smaller mitochondria. The main protein mediating mitochondrial fission is dynamin-related protein 1 (Drp1), whereas mitochondrial fission factor (Mff), fission mitochondrial 1 (Fis1), mitochondrial dynamics protein of 51 kDa (Mid51), and mitochondrial dynamics protein of 49 kDa (Mid49) act as receptors for Drp1 on the mitochondria, assisting in its recruitment to the mitochondria [42,43].

Drp1 is a key regulatory factor for mitochondrial fission and plays a major role in regulating mitochondrial dynamics. Additionally, the activation of Drp1 is a prerequisite for initiating the process of mitophagy [44]. After being activated by various signals, Drp1 translocates from the cytoplasm to the OMM, forming a ring-shaped polymeric structure at the fission site around the mitochondria. It causes the mitochondria to contract and break into daughter mitochondria by hydrolysing GTP in a GTPase—dependent way, thereby mediating mitochondrial fission [45]. The translocation and function of Drp1 are regulated by post-translational phosphorylation. Some viruses target Ser616 and Ser637 phosphorylation sites in Drp1 to regulate mitochondrial fission [46].

Mitochondrial fission plays a crucial role in maintaining the function of the mitochondrial network and regulating intracellular energy metabolism, cell proliferation, and apoptosis. Disrupting the interaction of proteins related to mitochondrial fission adversely affects mitochondrial dynamics and consequently cell function [47,48].

### 3.2. Viral Infection Regulates Alterations in Mitochondrial Dynamics

**Figure 1:**
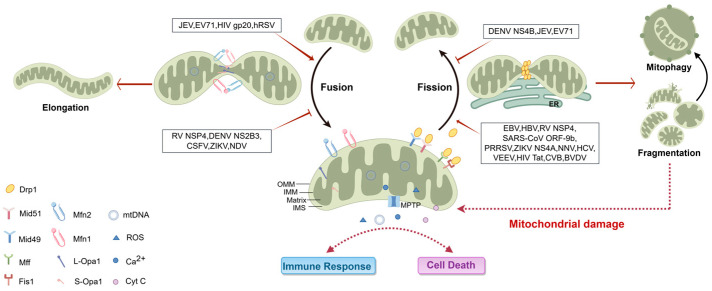
Mitochondrial dynamics processes and viruses or viral proteins regulating mitochondrial dynamics. The fusion of the OMM and IMM is respectively mediated by Mfn1/2 and L-Opa1/S-Opa1. Mitochondrial fission begins with ER-mediated mitochondrial contraction and the conformational change of Drp1 after being recruited to the fission site through OMM adaptor proteins (Mff, Mid49, Mid51, Fis1), which subsequently induces OMM rupture and generates mitochondrial fragments. Damaged mitochondrial fragments are cleared by mitophagy. Impaired mitochondrial fusion leads to mitochondrial elongation or accelerated mitochondrial fragmentation. The alternating process of mitochondrial fission and fusion is in a dynamic balance. Various viruses or viral proteins can disrupt this balance mechanism, for instance, by inhibiting the function of Mfn1/2 or Opa1 to hinder fusion or by overactivating Drp1 to promote fission, ultimately resulting in abnormal opening of the MPTP, releasing DAMPs such as mtDNA, ROS, Ca^2+^, and Cyt C, triggering mitochondrial-related immune responses and altering cell death outcomes.

Viruses manipulate mitochondrial dynamics, disrupt the balance between mitochondrial fission and fusion, and alter mitochondrial dynamics-mediated functions to achieve continuous proliferation [49]. Different viruses affect mitochondrial dynamics through different mechanisms. Owing to the constant pressure from the host immune system, viruses develop different strategies to counteract the host’s immune response. Viral proteins such as regulatory proteins, structural proteins, non-structural proteins, or accessory viral proteins can evade apoptosis, promoting the survival of infected cells, and use alterations in mitochondrial dynamics to facilitate viral replication (Figure 1). Viruses induce mitochondrial fission by reducing the expression of mitochondrial fusion proteins or increasing the expression of mitochondrial fission proteins, leading to mitochondrial dynamics disorders with a tendency towards fission. This results in mitophagy, disruption of interferon production, and control of the apoptotic pathway. The increase in mitochondrial number due to mitochondrial fission “works for the virus”, providing energy for the assembly of new viral particles to support long-term viral infection. Epstein–Barr virus (EBV) latent membrane protein 2A (LMP2A) induces Drp1 upregulation through the Notch signalling pathway, leading to enhanced mitochondrial fission in gastric and breast cancer cells, indicating that LMP2A protein regulates mitochondrial dynamics and plays a major role in EBV-related epithelial cancers [50]. HBV and hepatitis C virus (HCV) induce mitochondrial fission during infection to suppress the innate immune response and apoptosis and promote viral replication. The expression levels of Drp1 and its receptor Mff are both upregulated [51]. HCV further induces Drp1 (Ser616) phosphorylation, causing its translocation to mitochondria and inducing mitophagy [52]. Additionally, some viruses inhibit mitochondrial fusion by downregulating the expression of mitochondrial fusion genes such as *Mfn*, leading to mitochondrial fragmentation and promoting viral replication. For example, human respiratory syncytial virus (RSV) infection causes mitochondrial cristae shortening, matrix swelling, membrane damage, and mitochondrial elongation. The mRNA levels of mitochondrial fusion proteins *Mfn* and pore-forming proteins such as *VDAC2* are decreased, suggesting alterations in mitochondrial dynamics and obstruction of mitochondrial fusion, which may induce mitophagy to engulf damaged mitochondria [14]. Classical swine fever virus (CSFV) and zika virus (ZIKV) also cause mitochondrial fragmentation by downregulating Mfn2 expression, resulting in a lack of mitochondrial fusion [53,54].

Some viruses disrupt mitochondrial dynamics by mediating mitochondrial fusion. For example, the gp120 protein of human immunodeficiency virus (HIV) induces Mfn1 expression and reduces Drp1 expression, thereby promoting mitochondrial fusion and causing neuronal dysfunction and neurodegeneration [55]. Infection with Japanese encephalitis virus (JEV) affects mitochondrial fission and fusion simultaneously. After infection, the mitochondrial copy number in cells decreases. The activation of mitochondrial fission genes (*Fis1*/*Drp1*) decreases, whereas the activation of mitochondrial fusion genes (*Mfn1*/*Mfn2*/*Opa1*) increases. These genes are associated with increased ROS production and neuronal cell death after JEV infection [56]. Enterovirus 71 (EV71) infection in human neuroblastoma cells inhibits Drp1-related mitochondrial fission and enhances Mfn2-mediated fusion, leading to abnormal mitochondrial dynamics. The restoration of this function has an antiviral effect [57].

Different proteins of the same virus or the same protein expressed by different genotypes of the virus have different effects on mitochondrial dynamics and function and thus have different effects on cells. Dengue virus (DENV) infection induces impairment of mitochondrial fusion. The DENV protease NS2B3 cleaves Mfn1 and Mfn2 to inhibit mitochondrial fusion and exacerbate DENV-induced cytopathic effects [58], whereas the DENV non-structural protein NS4B disrupts mitochondrial dynamics by inhibiting Drp1 activation and promoting mitochondrial fusion and elongation, thereby creating conditions favourable for DENV replication [59]. This difference in effect may be due to differences in viral load and duration of infection, which cause these two proteins to have different effects on mitochondrial dynamics. The mechanisms by which viruses induce changes in mitochondrial dynamics may vary at different stages of infection. Additionally, in their study on hepatitis B virus X (HBx) protein-induced mitochondrial structural and functional abnormalities, Schollmeier et al. [60] found that the changes in mitochondrial dynamics and function mediated by HBx depended on the HBV genotype. Genotypes A and G of HBx caused more severe damage to the mitochondrial network structure than those of genotypes B, E, and C.

### 3.3. Mitochondrial Dynamics Mediate Mitochondrial Antiviral Responses to Viral Infection

In addition to serving as energy sensors and metabolic signalling centres, mitochondria also function as hubs for the innate immune response, regulating the body’s antiviral immune response. Viral infection can induce the innate immune response through mitochondrial dynamics to regulate the body’s antiviral mechanisms [61]. The role of mitochondrial dynamics in innate immune signalling is an emerging field of research.

The innate immune response is the first line of defence against viral infection. It recognises pathogen-associated molecular patterns (PAMPs) through pattern recognition receptors (PRRs) of the innate immune system and initiate processes to eliminate potential infections. PRRs include five families: toll-like receptors (TLRs), nucleotide-binding oligomerisation domain (NOD)-like receptors, C-type lectin receptors, retinoic acid-inducible gene I (RIG-I)-like receptors (RLRs), and inflammasomes. PRRs control the expression of pro-inflammatory cytokines, chemokines, and co-stimulatory molecules, creating an environment that helps cells resist viral infection and activates adaptive immunity [62,63]. For example, during some types of RNA virus infections, PAMPs are recognised by TLRs and RLRs, activating adaptor proteins such as RIG-I through caspase activation and recruitment domain (CARD) structures to recruit and activate downstream MAVS. The MAVS located in the cytoplasm or OMM are recruited and concentrated in the OMM region [64]. MAVS also acts as a platform to promote the interaction between adaptor proteins and downstream molecules such as interferon regulatory factor and nuclear factor-κB (NF-κB), which, on activation, enter the nucleus to induce the expression of type I interferons (IFN-I) and other inflammatory factors to exert antiviral effects [65,66]. In DNA viral infections, such as varicella-zoster virus (VZV) infection, DNA released in the cytoplasm is recognised by DNA sensors such as stimulator of interferon genes (STING), activating TANK-binding kinase 1 (TBK1) and leading to MAVS activation to further transmit antiviral signals [67], highlighting the importance of the MAVS pathway in the innate immune response induced by viruses in mitochondria. Besides DNA viruses, STING can also be activated by mtDNA released into the cytoplasm due to mitochondrial dynamics disorder caused by viral infection, mediating a series of innate immune responses. The main pathway is the cyclic guanosine monophosphate-adenosine monophosphate (cGAMP) synthase (cGAS)-STING pathway. cGAS is a sensor that can recognize mtDNA in the cytoplasm. After binding to mtDNA, cGAS is activated and catalyses the production of cGAMP. cGAMP acts as a second messenger to bind and activate STING, thereby increasing the expression of immune molecules such as IFN [68]. mtDNA can also be recognized by PRRs, such as Toll-like receptor 9 (TLR9), which is one of the important receptors for recognizing mtDNA. When TLR9 binds to mtDNA, it activates downstream signaling cascades, recruits myeloid differentiation factor 88 (MyD88), and subsequently activates transcription factors such as NF-κB and IRFs to enhance the expression of immune molecules such as IFN-I and improve antiviral and immune regulatory functions [69]. The production of mtDNA also involves the action of viral proteins, such as the viroporin activity of influenza virus M2 or EMCV 2b proteins, which is essential for the release of mtDNA into the cytoplasm. In addition, the influenza virus NS1 protein can bind to mtDNA to weaken cGAS-dependent immune responses [68]. Moreover, mtDNA can be recognized as mtDAMPs by inflammasomes such as NOD-like receptor family pyrin domain-containing 3 (NLRP3), absent in melanoma 2 (AIM2), and Z-DNA binding protein 1 (ZBP1), promoting the maturation and release of pro-inflammatory cytokines such as interleukin-1β (IL-1β) and interleukin-18 (IL-18). Meanwhile, IFN-I mediated by MAVS in innate antiviral immunity can regulate the activity of inflammasomes and recruit them to mitochondria, generating a synergistic immune response [70]. It is evident that mitochondria play a crucial role in mediating innate immune responses during viral infections.

Mitochondrial dynamics play a regulatory role in this intrinsic defence mechanism. The number of PRRs is directly related to mitochondrial dynamics. Mitochondrial fusion and fission play a core role in amplifying or inhibiting RLR signalling. Many viruses disrupt MAVS signalling through mitochondrial dynamics to impair the host immune response and promote their own replication. In the study of sendai virus (SEV), the activation of RLRs promoted the extension of the mitochondrial network and enhanced downstream signalling of MAVS and facilitated the binding of MAVS and STING. However, mitochondrial fragmentation inhibited this signalling and reduced the association between the two proteins. The study also revealed that MAVS is related to the mitochondrial fusion protein Mfn1, indicating that MAVS regulates mitochondrial dynamics and promotes the association between mitochondria and the endoplasmic reticulum required for signal transduction [71]. The above research content was further deepened in another study. SEV infection led to the redistribution of MAVS, and the mitochondrial fusion protein Mfn1 could interact with MAVS on the OMM and positively regulate the RLRs-mediated innate antiviral response. Knockout of Mfn1 led to the failure of MAVS redistribution and the inability to induce IFN to exert its antiviral effect, indicating that mitochondria may be involved in the regulation of MAVS through the fusion process [72]. Additionally, MAVS signalling activated by SEV infection promotes the activation of the inflammasome NLRP3. MAVS facilitates the recruitment of NLRP3 to mitochondria and may enhance its oligomerization and activation by bringing it closer to mitochondrial reactive oxygen species, thereby strengthening the immune response to SEV infection [73]. Koshiba et al. [74] confirmed that the deletion of mitochondrial fusion proteins Mfn1 and Mfn2, which causes mitochondria to lose the ability to fuse, leads to the failure of IFN and pro-inflammatory cytokine induction, thereby promoting viral replication. In the study of DENV infection, Yuet et al. [58] also revealed that Mfn1 is essential in the antiviral RLRs signalling pathway. Knockout of Mfn1 blocked mitochondrial fusion and weakened RLRs signalling, enhancing DENV infection. The above studies fully demonstrate that mitochondrial fusion promotes the interaction between MAVS and downstream signalling molecules, inducing the innate immune response to inhibit viral replication. The study by Hanada et al. [75] found that, unlike mitochondrial fusion function, mitochondrial fission does not directly affect antiviral responses. Antiviral cytokines are induced under conditions of mitochondrial fission defects. The mitochondrial fission factor Mff plays a key role in regulating the distribution of MAVS on mitochondria, and Mff can also sense the energy state of mitochondria. Mff activates MAVS and adjusts its redistribution, a process independent of the mitochondrial fission process and the fission factor Drp1. When mitochondrial function is impaired, Mff is phosphorylated by the cellular energy sensor adenosine 5′-monophosphate (AMP)-activated protein kinase (AMPK), leading to the disorder of MAVS redistribution and the inhibition of acute antiviral responses [76], indicating that Mff senses mitochondrial energy metabolism through AMPK signalling and plays a key role in MAVS-mediated innate immunity. In addition to the functional role of Mff on MAVS, high expression of mitochondrial fission factors Drp1 or Fis1 weakens RLRs signalling and inhibits the response of MAVS. Viral infection can induce the activation of Drp1, thereby promoting mitochondrial fission and blocking the activation of the MAVS-mediated innate immune response. In HCV infection, the virus mediates the upregulation of Drp1 expression, promoting mitochondrial fission and inhibiting the innate immune response to achieve continuous viral replication. Conversely, the absence of Drp1, enhances IFN signalling and inhibits the release of HCV [51]. SARS-CoV-2 open reading frame 9b (ORF-9b) acts on mitochondria and causes mitochondrial elongation by ubiquitinating Drp1 and proteasomal degradation. The ORF-9b protein can also bind to MAVS, promoting k48-linked ubiquitination-dependent proteasomal degradation of MAVS, thereby interfering with MAVS downstream signalling and IFN production [77]. Infection of mouse fibroblast L929 cells with ectromelia virus (ECTV) revealed that the alteration of mitochondrial network morphology regulates MAVS-dependent innate immunity, and mitochondrial network fragmentation inhibits the MAVS-mediated immune response [78]. In ZIKV-infected JEG-3 trophoblast cells, its non-structural protein 4A (NS4A) translocates to the mitochondria, triggering mitochondrial fission and inhibiting MAVS-mediated IFN responses [79]. The EBV-encoded BCL2 homolog 1 (BHRF1) protein, a viral homolog of BCL2, disrupts mitochondrial dynamics by targeting mitochondria, promoting Drp1-mediated mitochondrial fission and inhibiting MAVS-mediated IRF3 and IFN-I innate immune responses [80]. Similarly, in fish diseases, such as nervous necrosis virus (NNV) infection, virus-induced oxidative stress leads to Drp1 phosphorylation and mediates mitochondrial fragmentation, thereby inhibiting MAVS signalling in sea bass cells [81]. One possible reason for the inhibition of mitochondrial innate immune signalling due to mitochondrial fragmentation caused by the above-mentioned viral infections is that mitochondrial fragmentation triggers mitophagy to clear damaged mitochondria, thereby suppressing the innate immune response [82]. Signal transducer and activator of transcription 2 (Stat2) plays a key role in promoting Drp1 phosphorylation at the S616 site, inducing mitochondrial fission and maintaining mitochondrial homeostasis. After viral infection, Stat2 translocates to mitochondria and is degraded by viral disintegration formed by viral proteins and MAVS. Alongshan virus (ALSV) non-structural protein 1 (NSP1) binds to Stat2 through F175/R176 sites, leading to degradation of Stat2. Fis1 and Drp1 levels are decreased in a dose-dependent manner, the downstream IFN-I signalling pathway is disrupted, and the expression of interferon-stimulated genes is inhibited in order to inhibit the innate immune response [83]. These studies fully demonstrate that mitochondrial dynamics regulate MAVS antiviral signalling, and the integrity of the mitochondrial network and continuous respiratory capacity are necessary for early antiviral innate immune response.

### 3.4. Mitochondrial Dynamics Regulate Apoptosis After Viral Infection

**Figure 2:**
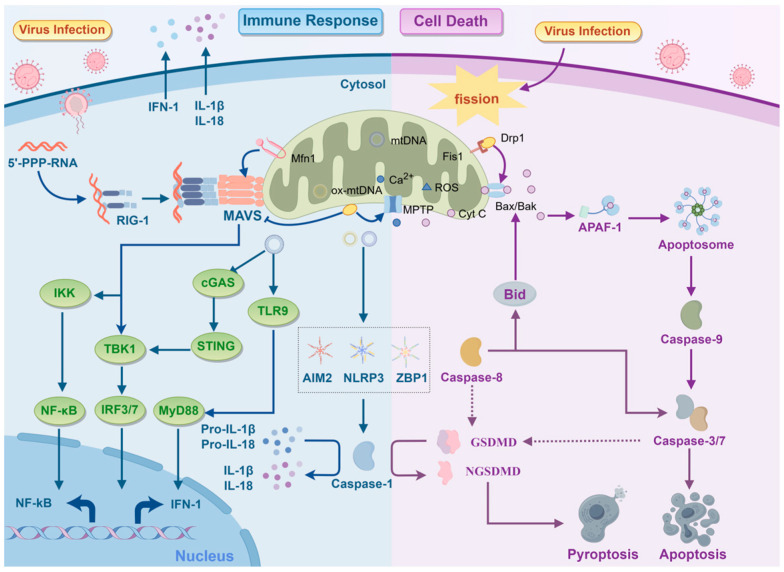
Viral infection triggers alterations in mitochondrial dynamics and affects the host immune response and cell death patterns. RIG in host cells can sense viral RNA and induce the aggregation of MAVS on the OMM, further triggering NF-κB and IFN-1 responses. Mfn1 maintains the integrity of the mitochondrial network, promoting MAVS aggregation and signal transduction; Drp1, on the other hand, causes excessive mitochondrial fission, dispersing MAVS and reducing signal transduction efficiency. Viral-mediated mitochondrial dynamics imbalance (such as fusion inhibition or excessive fission) leads to the opening of the MPTP, releasing mtDNA, ROS, and other DAMPs, triggering inflammatory responses. For instance, cytoplasmic mtDNA is recognized by cGAS, activating the STING-TBK1-IRF3 axis or the TLR9-MyD88 axis, promoting IFN-I production; ox-mtDNA activates NLRP3/AIM2/ZBP1 inflammasomes, activating Caspase 1, cleaving GSDMD to form plasma membrane pores, releasing IL-1β/IL-18, and triggering pyroptosis. Drp1-mediated mitochondrial fission promotes the oligomerization of pro-apoptotic proteins Bax/Bak, facilitating the release of Cyt C and activating the Caspase cascade, initiating apoptosis. In summary, viruses achieve immune evasion and death mode conversion by disrupting the balance of mitochondrial dynamics (inhibiting fusion proteins Mfn1/2 and activating fission protein Drp1).

Both the innate immunity of host cells and apoptosis are crucial host antiviral defence mechanisms. However, many viruses have evolved various strategies to manipulate apoptosis and evade the host’s antiviral immune response (Figure 2). Viruses may either upregulate anti-apoptotic genes to resist apoptosis or stimulate apoptosis to promote the shedding and spread of viral particles while preventing antigen presentation, depending on the stage of viral infection [84]. Viruses inhibit apoptosis in the early stage of infection to prevent premature cell death, but in the later stage, they induce apoptosis in host cells to release and spread the virus.

Apoptosis, also known as programmed cell death, is divided into the endogenous mitochondrial-mediated apoptotic pathway and the exogenous death receptor pathway, which both play a crucial role in maintaining normal cellular physiological functions. The endogenous apoptotic pathway is mediated by mitochondria. Under the influence of intracellular stress signals, the OMM is regulated by pro-apoptotic and anti-apoptotic factors of the BCL-2 family. The pro-apoptotic BH3 subfamily inhibits the function of anti-apoptotic BCL-2 proteins through protein–protein interactions [85]. Under apoptotic signal stimulation or viral infection, BCL-2-associated X protein (Bax) and BCL-2 antagonist killer (Bak) are activated, inducing changes in mitochondrial membrane permeability and the release of several pro-apoptotic factors such as cytochrome c (Cyt C) from the mitochondria into the cytoplasm, participating in the caspase cascade reaction. First, Cyt C combines with apoptotic protease activating factor-1 (APAF-1) and caspase-9 to form an apoptosome, activating caspase-9, leading to the activation of downstream effector caspases such as caspase-3, caspase-6, and caspase-7 and destruction of the cytoskeleton, inhibition of DNA repair, initiation of DNA fragmentation, and exposure of “eat-me” signals, which mediate the uptake and elimination of apoptotic cells by macrophages [86,87]. The apoptotic signals of the exogenous apoptotic pathway usually come from outside the cell and are caused by the interaction of extracellular death ligands with death receptors on the cell surface. Their binding recruits and activates adaptor proteins, such as Fas-associated protein with a novel death domain (FADD). FADD recruits and activates caspase-8 through its death effector domain [88]. In some cell types, activated caspase-8 directly activates downstream effector caspases such as caspase-3, triggering apoptosis [89]. However, in some other cell types, caspase-8 cleaves the BH3-interaction domain death agonist (BID) protein to generate truncated BID, which can be transferred to the mitochondria, releasing Cyt C and activating Bax and Bak in the intrinsic apoptotic pathway, thereby connecting the intrinsic and extrinsic apoptotic pathways. This phenomenon is known as “death receptor-mediated mitochondrial apoptotic pathway activation” [90].

Mitochondrial dysfunction, especially mitochondrial dynamics disorder, is closely connected to apoptosis. Once mitochondrial dynamics are disrupted, an unhealthy mitochondrial pool is produced, leading to energy deficiency, inducing apoptosis [91]. Viruses can induce mitochondrial dynamics disorder directly through viral proteins or by causing mitochondrial damage to reduce MMP or accumulate ROS, thereby increasing mitochondrial fission levels. Mitochondrial fission enhances the sensitivity of cells to apoptotic stimuli, whereas an increase in mitochondrial fusion levels is negatively correlated with Bax activation, Cyt C release, and cell death [92]. Mitochondrial fusion protects cells from apoptosis. Because Cyt C is located on the outer side of IMM, it is retained within the mitochondria through the action of Opa1. Opa1 plays an anti-apoptotic role by reshaping the mitochondrial cristae and maintaining their tight connection during apoptosis. Although Opa1 does not interfere with the activation of mitochondrial apoptosis regulatory factors Bax and Bak, it prevents apoptosis by reducing the release of Cyt C induced by mitochondrial membrane permeability [93,94]. The relationship between mitochondrial fission and apoptosis depends on Drp1, which induces mitochondrial fission and aggregation on the OMM under different stresses. During mitochondrial fission, the change in membrane permeability leads to the release of apoptotic factors, promoting Bak oligomerisation and insertion into the mitochondria and interaction with Bax. These apoptotic factors colocalise on the mitochondria, mediating mitochondrial remodelling and promoting the transport of pro-apoptotic proteins such as Cyt C and apoptosis-inducing factor to the cytoplasm, activating the key downstream effector caspase-3 in the apoptotic pathway, and triggering mitochondrial-mediated apoptosis [95,96]. In addition, mitochondrial Ca^2+^ overload promotes apoptosis through the binding of Drp1 and Bax, mainly because ERMC regulates type 1 inositol 1,4,5-trisphosphate receptor (IP3R1) and the VDAC1–mediated cell death pathway. IP3R1 and VDAC1 interact and serve as calcium channels, directly transferring calcium from the ER to the mitochondria. When ERMC is inhibited, the amount of Ca^2+^ in the mitochondria is reduced, delaying apoptosis. DENV and ZIKV promote viral replication through this pathway [97]. When the Drp1 inhibitor mitochondrial division inhibitor 1 (Mdivi-1) inhibits this process, it blocks mitochondrial fission, inhibiting the release of Bax/Bak and Cyt C and suppressing apoptosis [98]. For example, the attenuated live vaccine strain TC-83 of Venezuelan equine encephalomyelitis virus (VEEV) causes ROS accumulation and mitochondrial damage in astrocytoma cells, recruits Drp1 to mitochondria to induce mitochondrial fission, and Mdivi-1 inhibits Drp1 activity to suppress the caspase cascade reaction, demonstrating that mitochondrial fission contributes to apoptosis [99]. The NSP4 protein of rotavirus (RV) interacts with Drp1 to promote mitochondrial fragmentation. Drp1 inhibitors Mdivi-1 and RO-3306 inhibit the release of Cyt C into the cytoplasm and significantly degrade caspase-9 and caspase-3, resulting in a decrease in the level of mitochondrial-mediated apoptosis [100]. Apoptosis also regulates mitochondrial fission during viral infection. In the process of apoptotic cell death, pro-apoptotic factors Bax/Bak promote the ubiquitination of Drp1, stabilising Drp1 on mitochondria and promoting mitochondrial fission, achieving a synergistic effect between mitochondrial fission and apoptosis [101]. Additionally, excessive mitochondrial fission increases mitochondrial permeability transition pore permeability, promoting the release of Cyt C from mitochondria and leading to mitochondrial-mediated apoptosis [102,103]. However, Drp1-deficient cells also release Cyt C during the induction of apoptosis, indicating that mitochondrial fission and OMM permeability are mutually independent [52]. The upregulation of mitochondrial fission induced by viruses leads to mitophagy to clear damaged mitochondria and inhibit apoptosis, promoting the replication of viral particles. For example, HCV infection promotes mitochondrial fission and stimulates the expression of Drp1, phosphorylates the S616 site of Drp1 and causes its translocation to mitochondria to induce mitophagy, inhibiting apoptotic proteins such as Cyt C and caspase-3 and leading to persistent HCV infection [104]. Similar mechanisms are also found in bovine viral diarrhoea virus (BVDV), CSFV, and PRRSV [53,82,105]. Currently, the exact role of mitochondrial fission in apoptosis remains unclear.

Moreover, apoptosis-related molecules such as the caspase family and Bax/Bak, in addition to mediating apoptosis, also regulate another form of programmed cell death—pyroptosis—which is closely related to the inflammatory response. Pyroptosis mainly depends on caspase-1, and the latest research indicates that it also depends on caspase-3, caspase-6, caspase-7, and caspase-8, as well as mouse caspase-11 and its human homologs caspase-4 and caspase-5. Caspases specifically cleave Gasdermin D (GSDMD) at the interdomain loop and release the N-terminal pore-forming domain, which is then transferred to the plasma membrane to form a pore composed of 16 symmetric protomers with an inner diameter of 10–14 nm, leading to changes in cell osmotic pressure, cell swelling, and ultimately cell membrane rupture and release of intracellular contents, triggering a strong inflammatory response and causing pyroptosis [106]. Due to mitochondrial dynamics disorders leading to mitochondrial damage and the release of a large amount of ROS and mtDNA, they combine and activate inflammasomes such as NLRP3, recognize PAMPs and DAMPs, recruit and activate caspase-1, etc., thereby leading to the release of pro-inflammatory cytokines IL-1β and IL-18 and mediating pyroptosis. Bax/Bak activates caspase-3 and caspase-7 and subsequently induces K+ efflux to activate NLRP3 inflammasome and IL-1β secretion [107]. This process is also regulated by Drp1, which mediates mitochondrial fission and triggers NLRP3 inflammasome activation [108]. The above mechanism also exists in viral infection diseases, such as the non-structural protein NSs of RVFV triggering mitochondrial damage and activating NLRP3 inflammasome. The host transcriptional inhibition effect of NSs leads to rapid downregulation of the pro-survival protein myeloid cell leukemia-1 (MCL-1) in the BCL2 family, resulting in Bak activation in mitochondria, thereby triggering mtROS production and releasing oxidized mitochondrial DNA (ox-mtDNA) into the cytoplasm to combine and activate NLRP3 inflammasome, triggering NLRP3-GSDMD pyroptosis in RVFV-infected cells [109]. There is an interaction between the activation of the inflammasome and apoptosis. This interaction allows pyroptosis and apoptosis to be finely regulated according to the different stages and degrees of viral infection so as to achieve the best antiviral effect and minimum body damage.

### 3.5. Antiviral Therapeutic Mechanisms Targeting Mitochondrial Dynamics

Targeting the mechanism by which viruses use mitochondrial dynamics to regulate their own replication may exert antiviral effects. After infecting cells, most viruses use a variety of cell resources for replication. Excessive mitochondrial fission disrupts the normal function and structure of mitochondria, weakens the innate immune response, and provides a favourable environment for viral replication. In contrast, inhibiting mitochondrial fission maintains the normal morphology and function of mitochondria, reduces the energy and material supply required for viral replication, and induces the innate immune response of cells to inhibit viral replication. Currently, research on antiviral drugs targeting mitochondrial dynamics focuses on both chemically synthesised drugs and natural drugs that inhibit mitochondrial fission.

Drp1 is a key protein in mitochondrial fission. Inhibiting Drp1 activity reduces mitochondrial fission and inhibits viral replication in various viral infection models. Mdivi-1 was initially screened from a chemical library as a mitochondrial fission inhibitor in yeast and is effective at inhibiting the GTPase activity of Drp1. It prevents Drp1 from being recruited to the mitochondrial membrane and oligomerising to form a ring structure, inhibiting the mitochondrial fission process. For example, in coxsackievirus B (CVB)-infected mouse cardiomyocytes HL-1, inhibits Drp1 activity with Mdivi-1 resulting in a reduction of the viral titre [110]. Dynamin-related protein 1 inhibitor 1 (Drpitor1), is a highly specific Drp1-targeting inhibitor designed based on the GTPase domain of Drp1 through structure-based drug design and computer simulation screening. Based on this, Drpitor1a, a homologous compound with the methoxy-methyl group removed and improved hydrolytic stability, was further optimised and screened. Drpitor1a has a stronger inhibitory effect on mitochondrial fission than that of Mdivi-1 and inhibits the production of mitochondrial ROS. However, its application in viral infections has received very limited attention to date [111]. Additionally, a Drp1 peptide inhibitor, P110, was designed based on the mechanism by which Drp1 and its main receptors Mff and Fis1 mediate mitochondrial fission. Specifically, the Drp1-Mff-mediated mitochondrial fission process is a physiological process that plays an important role in maintaining MQC. Inhibiting their binding leads to cytotoxicity. In contrast, the Drp1-Fis1 interaction mediates pathological mitochondrial fission. Therefore, selectively inhibiting pathological rather than physiological mitochondrial fission is beneficial for maintaining mitochondrial homeostasis. Based on this, P110 was designed to selectively inhibit the Drp1-Fis1 interaction to inhibit abnormal mitochondrial fission [112]. In studies on acute kidney injury, P110 reduces the translocation of Drp1 to mitochondria and disrupts Drp1-Fis1 interaction to reduce mitochondrial fission. Moreover, high-dose administration of P110 does not have any obvious toxic effects [113]. Thus, P110 not only achieves the goal of inhibiting mitochondrial fission but also has a protective effect, making it promising for research in various disease models and it is expected to show clinically important antiviral therapeutic effects. In addition to compounds that inhibit Drp1, ginsenoside Rg3, extracted from *ginseng*, has been found to enhance the inhibitory effect of HCV core protein on the expression of cytosolic p21 in HCV infection, thereby inhibiting the binding of cyclin-dependent kinase 1 (CDK1) and Drp1. CDK1 phosphorylates Drp1 and facilitates its translocation to the mitochondrial membrane to induce mitochondrial fission. Inhibition of CDK1-Drp1 binding leads to a decrease in Drp1 phosphorylation and mitochondrial translocation, thereby reversing HCV-induced mitochondrial fission and inhibiting persistent HCV infection [29].

Furthermore, targeting mitochondrial fusion to inhibit mitochondrial fission is also an effective approach to antiviral therapy targeting mitochondrial dynamics. Echinacoside (ECH), a phenyl ethanol-based natural polyphenol compound extracted from *Cistanche*, promotes mitochondrial fusion. Specifically, ECH allosterically regulates the conformation of the casein kinase 2 (CK2) α subunit (CK2α) to recruit basic transcription factor 3 (BTF3) and form a binary protein complex. Subsequently, the CK2α/BTF3 complex promotes the nuclear translocation of β-catenin, activates T-cell factor/lymphoid enhancer factor (TCF/LEF), and stimulates transcription of the mitochondrial fusion gene *Mfn2*. Knockdown of CK2α expression eliminates ECH-mediated mitochondrial fusion, indicating that ECH promotes mitochondrial fusion through CK2α [114]. Resveratrol (RES), a natural plant polyphenol, is used as an antiviral drug for treating ocular abnormalities caused by ZIKV. Dihydroorotate dehydrogenase (DHODH), a key enzyme located on the IMM, mediates de novo pyrimidine nucleotide synthesis. DHODH inhibition leads to depletion of the pyrimidine pool, upregulating mitochondrial fusion proteins and promoting intracellular changes such as mitochondrial elongation. DHODH inhibition by RES increases mitochondrial fusion and reduces fission, thereby inhibiting ZIKV infection. The antiviral effect of RES against ZIKV is thought to be due to its effect on DHODH [115,116]. This mechanism is similar to that of leflunomide (LF), a compound used to treat rheumatoid arthritis, which was found to block pyrimidine biosynthesis by inhibiting DHODH, thereby increasing the expression of Mfn1 and Mfn2 and promoting mitochondrial fusion [117]. Melatonin has a regulatory effect on mitochondrial dynamics and regulates mitochondrial fusion through multiple pathways, such as directly acting on mitochondrial fusion-related genes such as *Mfn1*, *Mfn2*, and *Opa1*, or promoting mitochondrial fusion by reducing calcium accumulation and eliminating ROS production. AMPK is also a way for melatonin to promote mitochondrial fusion. Additionally, melatonin activates the Yap–Hippo signalling pathway to promote Opa1-mediated mitochondrial fusion [118]. Zacharioudakis et al. [119] identified the compound MASM7, which promotes mitochondrial fusion, through in-depth biochemical and structural analysis of mitochondrial fusion molecules Mfn1 and Mfn2. MASM7 increases the aspect ratio of mitochondria, promotes conformational activation of Mfn and increases oligomerisation, making it very effective for promoting mitochondrial fusion. However, the antiviral effect of MASM7 requires further study.

Some RNA viruses promote their replication by inducing mitochondrial fusion, so timely promotion of mitochondrial fission can be an effective antiviral approach. Mito-C is a novel heterocyclic compound member targeting the NADP(H)-dependent electron transfer enzyme (NEET) protein family, which targets mitochondria and rapidly causes mitochondrial network fragmentation. Nutrient-deprivation autophagy factor-1 (NAF-1), a member of the NEET protein family, is an important regulator of mitochondrial dynamics, promoting the recruitment of Drp1 to ERMC sites. Mito-C targets NAF-1 to promote Drp1-induced mitochondrial fragmentation, inhibiting DENV-induced excessive mitochondrial network fusion and viral replication [120]. The PB1-F2 protein of influenza A virus H1N1 is also a mitochondrial fission factor. Under serum starvation conditions, it induces mitochondrial fragmentation in multiple viral infection cycles. By controlling experimental conditions and optimising the experimental protocol to reduce cellular stress in the environment, influenza A virus H1N1 was found to induce mitochondrial network elongation and reduce the number of ERMC sites [121]. Mito-C, as a mitochondrial fission inducer, inhibits influenza A virus H1N1 replication, and Mito-C enhances the innate immune response of infected cells in a RIG-I-dependent manner to exert its antiviral effect [122]. Considering that the ORF-9 of SARS-CoV-2 promotes mitochondrial network elongation and degrades MAVS signals to inactivate the RLR pathway for viral replication, Mito-C might reverse this process to exert an anti-SARS-CoV-2 effect [123]. Zidovudine (AZT), a key component of some antiretroviral therapy regimens used to treat HIV infection, and its active metabolite AZT-triphosphate, exert their antiviral effect by upregulating the expression of Drp1 and Mff, the translocation of Drp1, and downregulating the expression of Opa1 to induce mitochondrial fission–fusion imbalance [124].

In addition to directly regulating mitochondrial fission and fusion to exert antiviral effects, some studies have focused on mitochondrial dynamics mediating apoptosis. For example, in a study of the impact of porcine deltacoronavirus (PDCoV) on apoptosis in swine testis (ST) cells and the antagonistic effect of selenium nanoparticles (SeNPs) against PDCoV, SeNPs alleviated PDCoV-induced mitochondrial fission, reduced the release of Cyt C and the activation of caspase-9 and caspase-3, and significantly reduced the replication of PDCoV in ST cells. This provides experimental evidence for the development and clinical application of anti-PDCoV drugs [125].

## 4. Conclusions and Future Directions

Mitochondria are key organelles that determine the fate of cells and are the targets of most viral invasions of host cells. Viruses use various strategies to manipulate mitochondria to promote their own survival, with mitochondrial dynamics being the primary mechanism used. Understanding how viruses disrupt the balance of mitochondrial dynamics and regulate mitochondrial-mediated innate immunity and apoptosis to achieve self-replication is an important scientific issue that requires further research. First, host cells are sensitive to the microenvironmental changes caused by viral infection stimuli, and more potential molecules involved in regulating mitochondrial dynamics need to be identified. Second, viral proteins are diverse, and viruses may produce different effects at different stages of host cell infection. Therefore, more detailed studies of these mechanisms are required. Continuous exploration of the common pathways and cytokines in the regulation of mitochondrial dynamics by different viruses is essential to help develop new antiviral therapies.

By screening active regulators of mitochondrial fission and fusion proteins from the perspectives of structural biology, biochemistry, cell biology, and chemistry and further validating the potential of mitochondrial dynamics proteins as antiviral drug targets through multiple methods, breakthroughs have been made in research on targeting mitochondrial dynamics as a form of antiviral therapy. However, significant challenges remain in translating these experimental results into clinical studies. Most antiviral drug research is based on in vitro cell models, whereas in vivo viral infections typically involve complex molecular network interaction mechanisms, and the application of antiviral drugs in vivo remains unclear. To further advance the development of drugs targeting mitochondrial dynamics, large-scale, artificial intelligence-assisted drug screening is required to expand the drug library targeting mitochondrial dynamics.

## Abbreviations

The following abbreviations are used in this manuscript:

ART	Antiretroviral therapy
ATP	Adenosine triphosphate
AZT	Zidovudine
Bak	BCL-2 antagonist killer
Bax	BCL-2-associated X protein
BID	BH3-interaction domain death agonist
CARD	Caspase activation and recruitment domain
CDK1	Cyclin-dependent kinase 1
CSFV	Classic swine fever virus
Cyt C	Cytochrome C
DENV	Dengue virus
DHODH	Dihydroorotate dehydrogenase
Drpitor1	Dynamin-related protein 1 inhibitor 1
ECH	Echinacoside
ERMC	Endoplasmic reticulum-mitochondria contact
FADD	Fas-associated protein with a novel death domain
GTP	Guanosine triphosphate
HBV	Hepatitis B virus
HBx	Hepatitis B virus X
HCV	Hepatitis C virus
HIV	Human immunodeficiency virus
HSV-1	Herpes simplex virus 1
IFN	Interferon
IMM	Inner mitochondrial membrane
JEV	Japanese encephalitis virus
L-Opa1	Long subtype of Opa1
LMP2A	Latent membrane protein 2A
MAVS	Mitochondrial antiviral signalling protein
Mdivi-1	Mitochondrial division inhibitor 1
Mff	Mitochondrial fission factor
Mfn1	Mitofusin 1
Mid	Mitochondrial dynamics protein
MMP	Mitochondrial membrane potential
MQC	Mitochondrial quality control
mtDNA	Mitochondrial DNA
NAF-1	Nutrient-deprivation autophagy factor-1
NEET	NADP(H)-dependent electron transfer enzyme
NOD	Nucleotide-binding oligomerisation domain
OMM	Outer mitochondrial membrane
Opa1	Optic atrophy protein 1
ORF	Open reading frame
OXPHOS	Oxidative phosphorylation
PAMPs	Pathogen-associated molecular patterns
PRR	Pattern-recognition receptor
RES	Resveratrol
RIG-I	Retinoic acid-inducible gene I
RLR	RIG-I-like receptors
ROS	Reactive oxygen species
SARS-CoV-2	Severe acute respiratory syndrome coronavirus 2
SeNP	Selenium nanoparticle
SEV	Sendai virus
S-Opa1	Short subtype of Opa1
ST	Swine testis
Stat2	Signal transducer and activator of transcription 2
STING	Stimulator of interferon genes
TBK	TANK-binding kinase
TCF/LEF	T-cell factor/lymphoid enhancer factor
TLR	Toll-like receptors
VDAC	Voltage-dependent anion channel
ZIKV	Zika virus
